# Report of the Trustees and Principal of the Indiana Asylum for the Deaf and Dumb; with an Exhibit of Expenditure

**Published:** 1847-12

**Authors:** 


### ARTICLE XV.

*Report of the Trustees and Principal of the Indiana Asylum for the Deaf and Dumb;* with an exhibit of expenditure. Presented to the General Assembly, Dec. 6th, 1847. (From Mr. Brown, Principal.)

To our profession, which ranks second to no other class or body of men in the *performance* of works of benevolence, a document like this must possess peculiar interest, as it shows unexampled prosperity in an institution eminently benevolent in its character.

The number of pupils under instruction in this Institution, which is in Indianapolis, at the date of the report, as appears from a catalogue appended, was eighty.

The following extracts from the full and satisfactory report of Mr. Brown, the intelligent and devoted principal of the Institution, will, we are confident, prove highly interesting to our readers, in a psycological point of view, as pourtraying the unfortunate condition of the deaf mute, and thus showing the great achievment in the process by which the barriers that lock up his mind from a knowledge of the world are overcome, and the interrupted channels of communication provided with a substitute.

"Of the senses possessed by mutes, that of sight is, of course, the most important. Through it they gain a superficial knowledge, at least, of surrounding objects. Shut out, as they are, from intercourse with others, this knowledge comes to them in all its primitive simplicity. The more common operations of nature are observed, without inquiry as to a cause; or if the query is suggested, it is easily satisfied with the apparent or secondary. That the trees should grow, the fields be clothed in verdure, streams run on in their courses, men and animals live and move, all seems too natural to excite a thought as to causation. That this state of things did not always exist, or that it requires an author, never enters their minds. The course of the sun, however, not unfrequently attracts their attention. Sometimes they suppose him endowed with life and motion, by the rays sent from his burning face, warming, and again scorching the earth, with the men and animals on its surface. Some think it an immense ball of fire, which a kind being above the blue vault draws up to the zenith in the morning, lets down to the west in the evening, and then at night, when men sleep, rolls round beneath the horizon to the east, ready for another day's journey through the sky. The stars seem tapers set in the arch of night, or small openings through which the glorious light beyond darts down upon us; and, strange to tell, they sometimes regard them as the bright eyes of beautiful beings of the upper sphere. The rain appears to some caused by a strong man, who dips up the waters of distant rivers, and then allows them to descend upon us. The jar of the thunder, for they are of course insensible to its sound, seems perhaps occasioned by heavy carriages, rolling rapidly over the welkin; while the lightnings are sparks and streams of fire from the forge of some huge Vulcan above. The winds they sometimes think occasioned by the united breath of a vast multitude of men, assembled in some remote place.

"Should theologians seek, in the above incoherences, the idea of a God, they will be forced to the conclusion, that mutes are naturally polytheists.

"The rights of religion are to them incomprehensible. They, perhaps, see their parents, morning and evening, bow around the family altar; they imitate the example, but kneel before an unknown God. People assemble on the Sabbath, and go through certain ceremonies; the mute knows not why, unless it be to propitiate some great being above to protect them from wild beasts, or other dangers. They see the dead followed to the tomb by weeping relatives, and a numerous throng of friends. The tears, the saddened looks, the solemn prayers, impress them with a dread of death which shrouds the grave in the most fearful terrors. That the dead ever rise to a fairer world, enters not their beclouded minds. As death seems the end of being, the soul's immortality is utterly unimagined.

"Of right and wrong, they seem to have little or no idea, previous to all education; though education, in this particular, is often commenced with some success at home. Even the right of property is not understood by many, and

I have been assured by those whose veracity could not be doubted, that they have repeatedly, before instruction, taken that which did not belong to them, without the slightest compunction of conscience. They are often found ignorant of the duty of respecting parental authority, and are surprised when they are punished for a violation. The approbation or displeasure of others, seems in many instances to be the sole rule by which they regulate their acts, without any reference to a higher principle or power. It should, however, be stated, to the credit of the parents and friends of the deaf and dumb, that they generally so far succeed in teaching the elementary principles of morals, that they come to institutions impressed with a high regard for truth and for the rights of property. It may be readily inferred from these facts, that they have not the higher idea of God as the ultimate judge of right and wrong.

"Of written law, human or divine, they are of course totally ignorant. An uneducated mute is sometimes present when a court is assembled to try a criminal. He sees the grave men busily engaged at, he knows not what. He sees the criminal confined in prison, and is utterly unable to divine the reason; or, he supposes the prisoner has incurred the hatred of the grave men, and that they take this way to manifest their anger. That this is the administration of justice, equal, even-handed right, enters not his imagination.

"Of language, in our understanding of the term, they have no idea. Signs, they have, which, in their uncouth state, may convey a few plain thoughts; but to them, letters, words, and books mean nothing. Their own names are as great mysteries as Chinese characters are to us. Even the fact that they or others have names is unknown. And, so dissimilar is the order of ideas in their imperfect gesture language from any language spoken or written, that were the words in sentences, rendered successively into signs, they would know little of the general meaning. The arrangement is just as unnatural to them, as the words in the columns of a spelling-book are to children capable of speech."

The report proceeds to give a history of this kind of education—an account of the steps taken in teaching, which is clear and concise—a synopsis of that which is wanted to make the Institution what it should be, (and we are glad to see that he advocates its being made a free school,) and after an account of the origin of the Institution, closes as follows:

"At the darkest hour of her trial, her finances in almost hopeless depression, while the cold, unpitying finger of scorn was beginning to point at her hitherto fair escutcheon, and the startling, though scarce-breathed whisper was heard, "Indiana will repudiate!" it was at this time our noble State remembered her unfortunate children, the Deaf and Dumb, the Lunatic, and the Blind. She took them by the hand, and scorning to take the funds which others might claim, though locked in her own treasury, she taxed her citizens to raise a special, a sacred revenue for their benefit.

"How stands the case now? Her credit is redeemed. A building, even now erected, tells how she will house and care for the poor Lunatic; already has she gathered her Blind from all quarters of her extensive domain, and presents, only four years after its organization, an Institution actually educating a greater number of Mutes, in proportion to her population, than any other State in the Union."

